# Selective 7‑Azaindole Modulators Targeting Fyn and GSK-3β for Dual-Target Neuromodulation

**DOI:** 10.1021/acsmedchemlett.5c00728

**Published:** 2025-12-24

**Authors:** Haofeng Shi, Yinlong Li, Steven H. Liang

**Affiliations:** † Department of Radiology and Imaging Sciences, 1371 Emory University, 1364 Clifton Road, Atlanta, Georgia 30322, United States; ‡ Wallace H. Coulter Department of Biomedical Engineering, Georgia Institute of Technology and Emory University, Atlanta, Georgia 30332, United States

**Keywords:** Fyn proto-oncogene kinase, glycogen synthase kinase 3β, structure−activity relationship, neurodegenerative diseases

## Abstract

Fyn proto-oncogene kinase (Fyn) and glycogen synthase kinase-3β (GSK-3β) belong to distinct branches of the protein kinase (PK) superfamily. Fyn is a member of the Src family of tyrosine kinases, whereas GSK-3β is classified within the CMGC group of serine/threonine kinases. Both play critical roles in neurodegenerative processes, and their dysregulation has been implicated in disease progression. The development of Fyn and GSK-3β inhibitors has attracted increasing research attention. The design of multitarget inhibitors represents a promising, though underexplored, therapeutic strategy. A recent study reported a series of dual selective nanomolar inhibitors based on structure–activity relationship (SAR) optimization. In-depth profiling of the lead compound’s neuroprotective and modulatory properties establishes a foundation for the development of next-generation neuroregenerative therapeutics.

## Graphical abstract

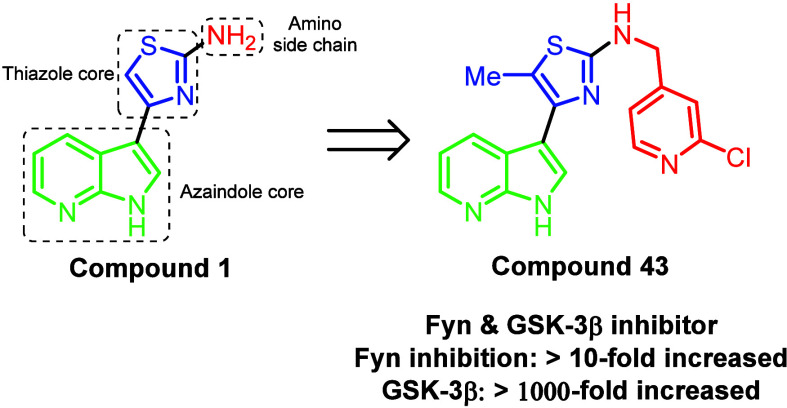

## Introduction

Protein kinases (PKs) catalyze protein phosphorylation that regulate cellular signaling through reversible protein modifications.^1,2^ Dysregulation of kinase activity is closely associated with the onset and progression of numerous human diseases.^3,4^ Based on catalytic domain homology, the human kinome is classified into eight major families.^5^ Among these, Fyn proto-oncogene kinase (Fyn), belonging to the Src subfamily of the tyrosine kinase (TK) family,^6^ and glycogen synthase kinase 3β (GSK-3β), a member of the CMGC family,^7^ are central regulators of cytoskeletal dynamics,^8^ metabolic homeostasis,^9,10^ and neuroplasticity^11^ via tyrosine phosphorylation^12^ and serine/threonine phosphorylation,^13,14^ respectively. Aberrant activation of Fyn and GSK-3β plays a pivotal role in neurodegenerative diseases such as Alzheimer’s disease (AD) and Parkinson’s disease (PD),^15,16^ establishing both kinases as key therapeutic targets for dual-modulation strategies.^17,18^

Fyn is widely expressed in the brain and participates in T-cell receptor signaling, brain function regulation, adhesion-related signaling, and cell survival.^19,20^ GSK-3β, predominantly expressed in the central nervous system (CNS), regulates cell division, proliferation, differentiation, and adhesion.^21,22^ Moreover, GSK-3β is closely associated with neuronal apoptosis, synaptic plasticity, axon formation, and neurogenesis.^23–26^ Given the multifactorial etiology and progressive nature of neurodegenerative diseases,^27^ current treatments primarily alleviate symptoms without altering disease progression.^28^ Research has shifted from single-target approaches^29,30^ toward multitarget intervention strategies that simultaneously suppress neuroinflammation and promote neuroregeneration.^31,32^ Within this framework, dual regulation of Fyn and GSK-3β represents a promising therapeutic avenue.^33^

Previous work on 7-azaindole-based molecules yielded potent Rho kinase (ROCK) and ROCK2 inhibitors with high inhibitory activity and favorable lipophilic ligand efficiency (LLE).^34,35^ Recent study revealed that derivatives of this scaffold exhibit moderate inhibitory effects against FYN and GSK-3β. Guided by systematic structural optimization of three key regions (Figure 1),^36^ modifications to the amino side chain were explored to enhance inhibitory activity through the incorporation of side chains with varying lengths and functional groups (e.g., aliphatic amines, aromatic amides). Among these analogs, compound **7**, featuring an *N*-benzyl substitution, exhibited improved inhibitory activity against Fyn and GSK-3β. Subsequent exploration involving substitutions on the phenyl ring or its replacement with *N*-heterocycles revealed that introduction of a 3-chloro-4-pyridyl moiety (compound **28**) yielded the first dual inhibitor with double-digit nanomolar potency (GSK-3β IC_50_ = 0.038 ± 0.006 μM; Fyn IC_50_ = 0.71 ± 0.09 μM). The modification of azaindole core was investigated via removal, substitution, or masking of either nitrogen atom, as well as introduction of other substituents onto the core scaffold. These results suggested the introduction of bromine atom at C-5 position markedly enhanced both selectivity and inhibitory potency toward Fyn (compound **38**, Fyn IC_50_ = 0.55 ± 0.02 μM). The following modification of thiazole core revealed introduction of a methyl group on the thiazole ring redirected the inhibitory profile toward Fyn (compound **39**, GSK-3β IC_50_ = 15.60 ± 3.47 μM; Fyn IC_50_ = 0.39 ± 0.11 μM). Integration of the most favorable structural fragments from each region yielded an optimized scaffold, compound **43**, with substantial improvements in potency and selectivity (GSK-3β IC_50_ = 0.61 ± 0.02 μM; Fyn IC_50_ = 0.044 ± 0.003 μM). This compound represents a promising dual-target inhibitor and a candidate for further neuroprotective and neuroregenerative investigation (Figure 1).

**Figure 1:**
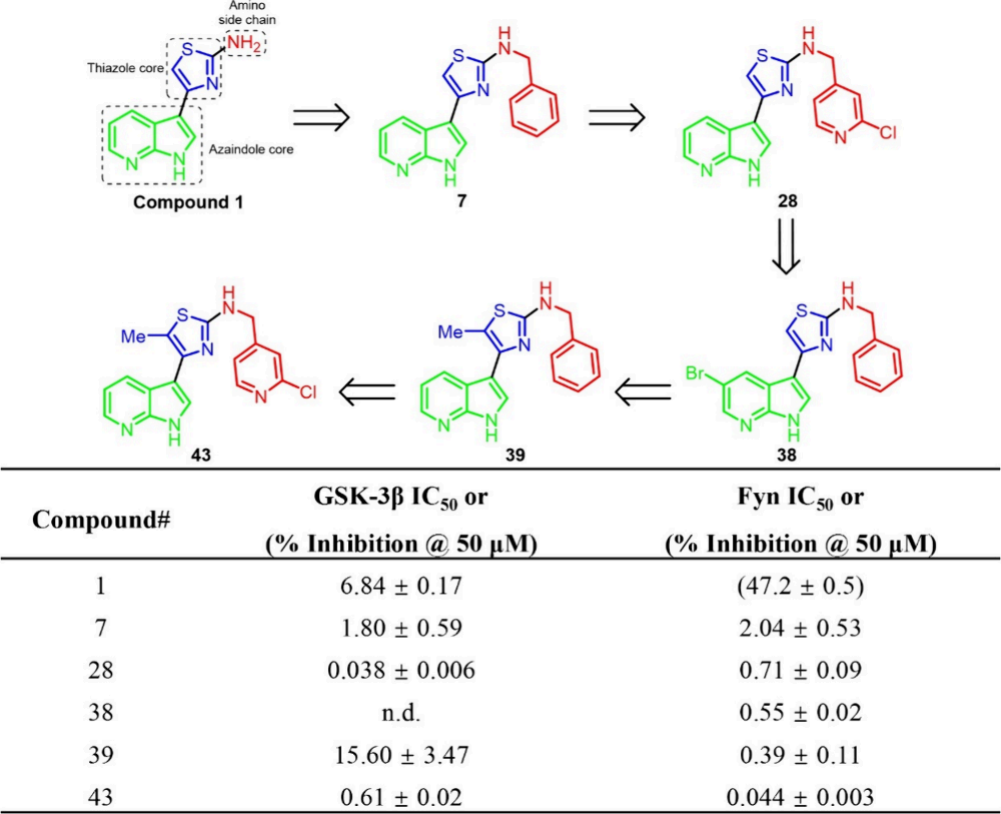
SAR optimization of Compound **1**. *^a^* Data are mean ± SD from ≥ 3 independent experiments, with IC_50_ values determined from dose–response curves for promising compounds. The data was adapted from ref 36. Copyright 2025 American Chemical Society.

Computational results revealed mechanistic insights into the binding interaction of compound **43** with Fyn and GSK-3β (Figure 2). For Fyn, compound **43** adopted a stable, fixed conformation within the binding pocket. The 5-methyl group of the thiazole ring was optimally positioned in a hydrophobic cavity, while the 7-azaindole moiety further stabilized the complex through hydrogen bonding with the backbone NH of Ser89. In contrast, when bound to GSK-3β, the 5-methyl group of the thiazole ring was sterically hindered by the Leu132 side chain, causing a slight displacement of the ligand from the binding pocket and attenuating halogen bonding and π–π interactions. This adverse spatial effect was partially compensated by a hydrogen bond between the pyridine ring of compound **43** and Arg141, which maintained its submicromolar inhibitory activity against GSK-3β.

**Figure 2:**
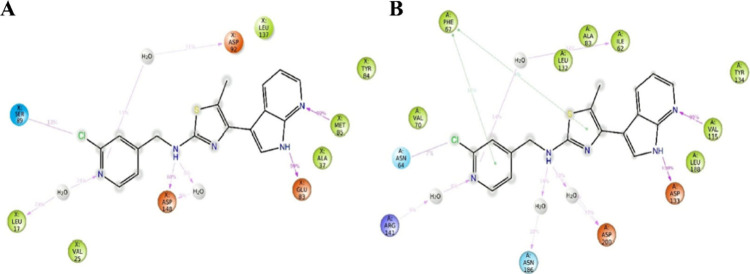
Bidimensional interaction profiles over time calculated on the last 50 ns of molecular dynamics simulation of **43** within Fyn (A) and GSK-3β (B). Green arrows are p–p interactions, red arrows are p–cation interactions, magenta lines are halogen bonds, and magenta arrow are H-bond interactions. The data was adapted from ref.^36^ Copyright 2025 American Chemical Society.

Biological evaluation demonstrated that compound **43** possesses multidimensional neuroactive properties and favorable drug-like characteristics. In primary rat cerebellar granule neuron (CGN) model, it showed no neurotoxicity at concentrations of 5–25 μM and slightly enhanced cell viability relative to the control. In the serum/potassium deprivation-induced neuronal senescence model, compound **43** completely reversed cell death and rescued neuronal viability, demonstrating a potent neuroprotective effect. In the mouse subventricular zone (SVZ) neurosphere model, drug concentrations of 0.1–5 μM failed to significantly promote neurosphere proliferation or maturation, in contrast to compounds with high GSK-3β inhibitory activity. However, in the neurosphere differentiation model, treatment with compound **43** at 1 μM selectively promoted astrocyte differentiation. In LPS-stimulated N9 microglia, compound **43** (2.5–5 μM) significantly downregulated the pro-inflammatory enzyme iNOS without altering anti-inflammatory TREM2 expression or microglial phagocytosis, supporting neuroinflammation alleviation via M1/M2 phenotypic switching. For blood-brain barrier (BBB) permeability, compound **43** exhibited an apparent permeability coefficient (Papp = 32 ± 5.38 × 10^–6^ cm/s) higher than positive controls (e.g., antipyrine, donepezil) and did not disrupt endothelial integrity at 100 μM, confirming favorable CNS penetration. Kinase profiling further demonstrated potent inhibition of Fyn (IC_50_ = 0.044 ± 0.003 μM) and GSK-3β (IC_50_ = 0.61 ± 0.02 μM), along with modulation of neurotoxic kinases Lyn/LOK/Abl, while engaging anti-inflammatory off-target kinases in a potentially synergistic manner.

## Future Outlook

Building on the promising findings of this study regarding 7-azaindole-based Fyn/GSK-3β inhibitors, compound **43** has emerged as the lead compound from a series of Fyn/GSK-3β inhibitors following an extensive SAR screening. Future studies should prioritize in vivo validation and pharmacokinetic/pharmacodynamic (PK/PD) optimization. Behavioral and neuropathological assessments in animal models of neurodegenerative diseases (e.g., AD transgenic mouse models) will be essential to confirm its efficacy in ameliorating cognitive impairments, reducing neurofibrillary tangles (NFTs), and restoring neuronal function. In parallel, the development of positron emission tomography (PET)^37,38^ tracers targeting Frn or GSK-3β separately is warranted to enable noninvasive evaluation of target engagement and dose–response relationships in living subjects.^39^ These studies will collectively advance the translational potential of dual Fyn/GSK-3β inhibition as a strategy for neuroprotective and neuroregenerative therapy.
